# Uncoupling of Akt and mTOR signaling drives resistance to Akt inhibition in PTEN loss prostate cancers

**DOI:** 10.1126/sciadv.adq3802

**Published:** 2025-02-07

**Authors:** Ninghui Mao, Young Sun Lee, Nazifa Salsabeel, Zeda Zhang, Dan Li, Harmanpreet Kaur, Xiaoping Chen, Qing Chang, Sanjay Mehta, Jesse Barnes, Elisa de Stanchina, Ralph Garippa, Yu Chen, Charles Sawyers, Brett S. Carver

**Affiliations:** ^1^ Human Oncology and Pathogenesis Program, Memorial Sloan Kettering Cancer Center, New York, NY 10065, USA.; ^2^ Department of Cancer Biology and Genetics, Memorial Sloan Kettering Cancer Center, New York, NY 10065, USA.; ^3^ Antitumor Assessment Core Facility, Memorial Sloan Kettering Cancer Center, New York, NY 10065, USA.; ^4^ The Gene Editing and Screening Core Facility, Memorial Sloan Kettering Cancer Center, New York, NY 10065, USA.; ^5^ Department of Medicine, Memorial Sloan Kettering Cancer Center, New York, NY 10065, USA.; ^6^ Howard Hughes Medical Institute, Memorial Sloan Kettering Cancer Center, New York, NY 10065, USA.; ^7^ Department of Surgery, Memorial Sloan Kettering Cancer Center, New York, NY 10065, USA.; ^8^ Division of Urology, Memorial Sloan Kettering Cancer Center, New York, NY 10065, USA.

## Abstract

Recent phase 3 clinical trial showed improved radiographic progression-free survival in PTEN-deficient prostate cancers treated with combined Akt and AR inhibition. Building on this and our previous research into PI3K and AR signaling interactions, we aimed to define the mechanisms of response and resistance to Akt inhibition. We discovered that restoration of mTOR signaling was the early dominant driver of resistance to Akt inhibition. Mechanistically, this can be achieved through molecular alterations, resulting in loss of negative regulators of mTOR. Unexpectedly, we discovered that this was dominated by restoration of mTOR signaling through the nutrient sensing arm. This can be achieved by loss of the components of the GATOR/KICSTOR complexes or through cellular processes, leading to the recycling of amino acids. The addition of an mTOR inhibitor restored sensitivity to Akt inhibition and represents a precision-based strategy to overcome resistance in the clinic.

## Teaser

Restoration of downstream mTOR signaling causes resistance to combined Akt and androgen receptor blockade in prostate cancer.

## INTRODUCTION

Molecular alterations leading to active phosphatidylinositol 3-kinase (PI3K) signaling (loss of *PTEN* and *PIK3CA/B* mutations) are among the most frequent events in metastatic prostate cancer occurring in greater than 60% of tumor specimens (*1*–*3*). While inhibition of androgen receptor (AR) is the main treatment for patients with metastatic hormone-sensitive prostate cancer and metastatic castrate-resistant prostate cancer (mCRPC), preclinical and clinical studies have demonstrated that loss of *PTEN* and aberrant PI3K signaling drive resistance to AR-targeted therapies (*4*, *5*). Thus, the PI3K signaling axis has been an attractive target for therapy in mCRPC, and multiple inhibitors targeting the various nodes of the pathway have been developed.

We previously defined the reciprocal inhibitory regulation between the PI3K and AR signaling pathways in the context of PTEN loss, such that inhibition of PI3K signaling resulted in hyperactive AR signaling, limiting response, and necessitating combined inhibition of PI3K and AR (*4*). Our work explained, in part, why initial clinical trials of single-agent PI3K/mammalian target of rapamycin (mTOR) pathway inhibitors were unsuccessful in mCRPC and led to the next generation of clinical trials combining PI3K and AR pathway inhibition (*4*, *6*). Recently, the randomized phase 3 clinical trial of the Akt inhibitor ipatasertib combined with the AR pathway inhibitor abiraterone acetate demonstrated a significant improvement in radiographic progression-free survival in PTEN-deficient mCRPC (*7*). Furthermore, a recent update from the clinical trial reported that patients harboring genomic alterations leading to active PI3K signaling (*PTEN* loss, *PIK3CA* mutation, and Akt mutation) had a greater than 6-month improvement in overall survival when treated with ipatasertib and abiraterone compared with abiraterone alone (*7*). Collectively, the data demonstrate that combined targeting of Akt and AR in PTEN-deficient mCRPC is therapeutically beneficial and that genomic classification of altered PI3K signaling may be the optimal selection criteria for Akt inhibitor therapy. Similarly, clinical trials in *PIK3CA*-mutant breast cancer have demonstrated that the combination of the PIK3CA-selective inhibitor alpelisib and estrogen receptor (ER) degrader fulvesterant improves radiographic progression-free survival (*8*).

Previous studies of response and resistance in prostate cancer preclinical models have been limited by a paucity of available cell lines. However, over recent years, the development of the prostate cancer organoid culture methodology has allowed us to establish a broad panel of patient-derived models (*9*). These models are representative of the genetic, histologic, and AR inhibitor responsive diversity observed in mCRPC (*9*). Collectively, the integration of established prostate cancer cell lines and patient-derived prostate cancer organoids allows us to define the questions of therapeutic response and resistance in a more rigorous fashion. Building on the recently completed and ongoing clinical trials of Akt and AR inhibition in prostate cancer, we have begun to define the mechanisms of response and resistance to Akt inhibition with the goal of optimizing patient selection and developing combinatorial and sequential therapeutic strategies to maximize response and overcome resistance.

## RESULTS

### Established models of PTEN loss display differential sensitivity to Akt inhibition

**Fig. 1.**
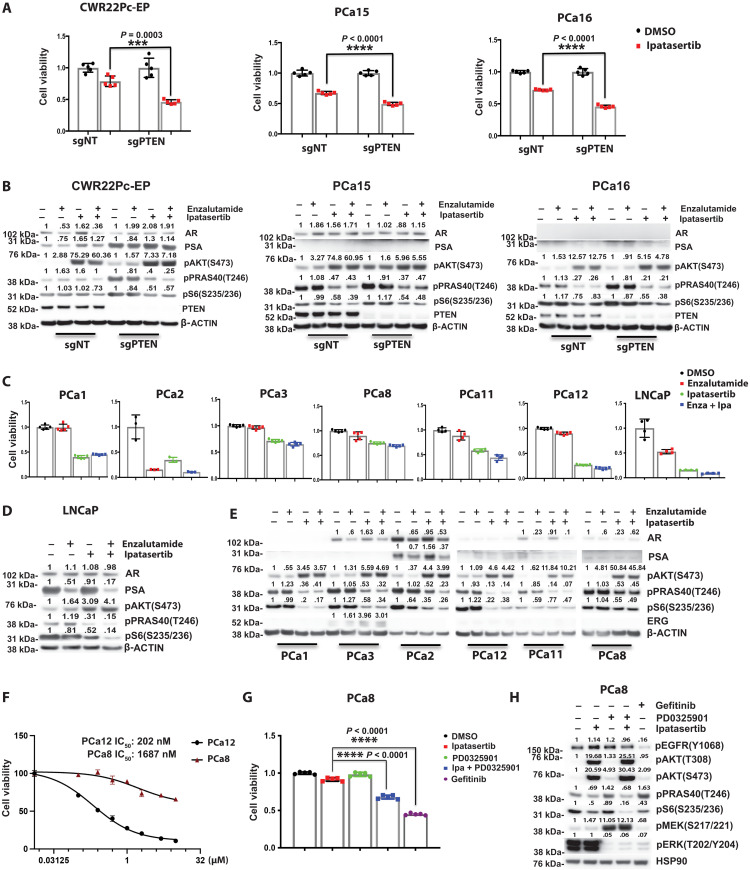
While PTEN loss enhances sensitivity to combined Akt and AR Inhibition, specific molecular alterations drive resistance in PTEN-deficient models. (**A**) Cell viability on day 7 in PTEN wild-type (WT) and CRISPR PTEN–modified CWR22Pc-EP, PCa15, and PCa16 treated with either ipatasertib (500 nM) or DMSO was assessed. (**B**) Expression levels of pS6, pPRAS40, and PSA were analyzed by Western blot in PTEN WT and CRISPR PTEN–modified CWR22Pc-EP, PCa15, and PCa16 cells. Cells were treated with DMSO, enzalutamide (10 μM) overnight, or ipatasertib (500 nM) for 4 hours. (**C**) Cell viability on day 7 was evaluated in PCa1, PCa2, PCa3, PCa8, PCa11, PCa12, and LNCaP following treatment with enzalutamide (Enza; 10 μM), ipatasertib (Ipa; 500 nM), or DMSO. (**D** and **E**) The expression levels of pS6, pPRAS40, and PSA in LNCaP, PCa1, PCa3, PCa2, PCa12, PCa11, and PCa8 were evaluated via Western Blot analysis. The cells were treated with DMSO, enzalutamide (10 μM) overnight, ipatasertib (500 nM) for 4 hours. (**F**) Cell viability on day 7 in PCa12 and PCa8, treated with various doses of ipatasertib, with the corresponding median inhibitory concentration (IC_50_) values displayed. (**G**) Cell viability on day 7 in PCa8 cells treated with the Akt inhibitor ipatasertib (500 nM), the MEK inhibitor PD0325901 (1 μM), the EGFR inhibitor gefitinib (1 μM), or DMSO. (**H**) The expression levels of downstream targets in the MAPK and PI3K pathways were assessed in PCa8 cells. Cells were treated with DMSO, the Akt inhibitor ipatasertib (500 nM), the MEK inhibitor PD0325901 (1 μM), and the EGFR inhibitor gefitinib (1 μM) for 4 hours [***P < 0.001 and ****P < 0.0001; (A and G) Welch’s *t* test; error bar represents ±SEM].

To evaluate the therapeutic effect of Akt inhibition in our preclinical mCRPC models, we first used isogenic pairs of CWR22Pc-EP, MSK-PCa15, and MSK-PCa16 to demonstrate that genomic loss of *PTEN* through CRISPR-Cas9 knockout resulted in enhanced sensitivity to ipatasertib (500 nM) in in vitro growth assays (Fig. 1A, fig. S1A, and table S1). Ipatasertib binds to the catalytic domain of Akt1/2/3 and inhibits the phosphorylation of Akt target proteins (*10*). Western blot analysis confirmed that loss of PTEN, activation of PI3K signaling, and Akt inhibition (ipatasertib, 500 nM; 4 hours) resulted in the down-regulation of the phosphorylation of Akt pathway proteins such as PRAS40 and S6 (Fig. 1B). In addition, ipatasertib is known to lock Akt in a hyperphosphorylated state, and this biochemistry can also serve as a marker for drug target engagement (*11*). In the AR-dependent CWR22Pc-EP model, which harbors an activating *PIK3CA* mutation, Akt inhibition led to an increase in AR target gene expression [prostate specific antigen (PSA)], which was suppressed by the AR inhibitor enzalutamide (10 μM, 12 hours). Notably, these experiments were designed to assess the immediate effects of target inhibition and were not intended to explore the cross-regulation between PI3K and AR signaling (Fig. 1B).

Next, we expanded our studies to determine whether there is differential sensitivity to combined Akt and AR inhibition across a broad panel of endogenous *PTEN*-deficient prostate cancer organoids and cell lines (table S1) (*9*, *12*). Briefly, all the organoids selected for the study were derived from patients with mCRPC with no evidence of neuroendocrine prostate cancer; harbor genomic loss of PTEN; and have adenocarcinoma histologic phenotypes ranging from more luminal to less luminal, variable expression of AR, and responsiveness to AR inhibition. To accomplish this, we performed in vitro growth assays following treatment with ipatasertib (500 nM) and/or enzalutamide (10 μM) (Fig. 1C). Western blot analyses demonstrated that acute inhibition of Akt with ipatasertib (500 nM, 4 hours) resulted in down-regulation of the Akt/mTOR pathway (Fig. 1, D and E). While we found that most of the *PTEN*-deficient models demonstrated sensitivity to Akt and/or AR inhibition, there was differential responsiveness across the models, and two model systems (PCa3 and PCa8) were inherently more resistant to Akt inhibition.

To identify potential mechanisms associated with innate resistance in our models, we analyzed the MSK Impact genomic profiling data available on our prostate cancer organoid models (MSK cBioPortal, www.cbioportal.org) (*9*). These studies revealed that PCa3 harbors a genomic rearrangement of *TMPRSS2:ERG*, leading to aberrant expression of ERG that we have previously shown promotes resistance to combined PI3K and AR pathway inhibition (*13*). PCa8, the most innately resistant *PTEN*-deficient model, harbors an intergenic fusion, leading to the expression of epidermal growth factor receptor variant III (EGFRvIII), and Akt inhibition resulted in a minimal impact on cell proliferation and downstream mTOR signaling (Fig. 1, E and F). Genomic alterations resulting in EGFRvIII expression have been reported in glioblastoma, promoting downstream MAPK and PI3K/Akt signaling, and display responsiveness to epidermal growth factor receptor (EGFR) inhibition with gefitinib or lapatinib (*14*, *15*). PCa8 was noted to have both active MAPK and PI3K signaling, and, while single-agent MEK (PD0325901, 1 μM) or Akt (ipatasertib, 500 nM) inhibition did not significantly affect cell proliferation, we hypothesized that the relief of feedback inhibition between the two pathways following single-pathway inhibition affected this (*16*). Single-agent MEK inhibition resulted in increased Akt activation, and single-agent Akt inhibition resulted in further MEK activation, suggesting that dual targeting of these pathways was required. Combined MEK and Akt inhibition resulted in inhibition of cell proliferation, and this was even more robust with the EGFR inhibitor gefitinib (1 μM) (Fig. 1, G and H). To validate that these cells were dependent on EGFRvIII signaling, we performed CRISPR-Cas9 knockout. We found that knockout of EGFR reduced downstream EGFR signaling, and the addition of ipatasertib further repressed mTOR signaling (fig. S1B). In addition, we found that knockout of EGFR suppressed cell proliferation, and the addition of ipatasertib enhanced this effect (fig. S1C). Collectively, our data demonstrate that, while loss of PTEN enriches for response to combined Akt and AR inhibition, there is inherent differential sensitivity across PTEN-deficient models, which is driven by concomitant molecular alterations.

### Restoration of mTOR signaling drives acquired resistance to Akt inhibition

**Fig. 2.**
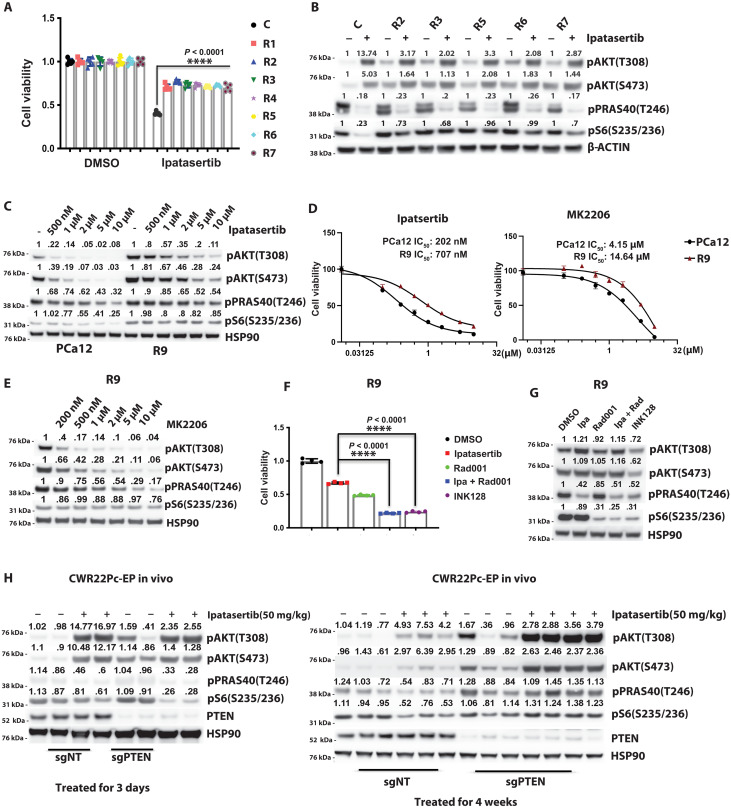
Restoration of mTOR signaling drives acquired resistance to Akt inhibition in vitro and in vivo. (**A**) Cell viability was assessed on day 7 in ipatasertib-resistant lines (R1 to R7), derived from PCa12 tumors treated with castration, enzalutamide (10 mg/kg), and ipatasertib (50 mg/kg) for 6 weeks. Control cells (C) were treated with castration and enzalutamide only and then cultured with enzalutamide (500 nM). Resistant lines were maintained with enzalutamide (500 nM) and ipatasertib (500 nM). (**B**) PI3K pathway targets were evaluated by Western blot in control (C) and resistant cells (R2, R3, R5, R6, and R7) after 4 hours of DMSO or ipatasertib treatment. (**C**) Western blot analysis of PI3K pathway targets in PCa12 and R9 cells treated with DMSO or ipatasertib at various doses for 4 hours. (**D**) Cell viability in PCa12 and R9 cells treated with ipatasertib or MK2206, with IC_50_ values shown. (**E**) Western blot of PI3K pathway targets in R9 cells after 4-hour treatment with vehicle or various dose of MK2206. (**F**) Day 7 cell viability in R9 cells treated with ipatasertib (500 nM), Rad001 (100 nM), INK128 (100 nM), or vehicle. (**G**) Western blot of PI3K pathway targets in R9 cells after treatment with DMSO, ipatasertib (500 nM), Rad001 (100 nM), or INK128 (100 nM) for 4 hours. (**H**) PI3K signaling was analyzed by Western blot in PTEN WT and CRISPR PTEN–modified CW22Pc-EP tumors treated with vehicle or ipatasertib (50 mg/kg) for 3 days or 4 weeks. Castration was performed when tumors reached ~300mm^3^ [****P < 0.0001; (A) one-way analysis of variance (ANOVA) versus control; (F) Welch’s *t* test; error bars, ±SEM].

To further study mechanisms of resistance associated with Akt inhibition, we sought to define the phenotypes emerging during the development of acquired resistance to combined AR and Akt inhibition. To achieve this, an in vivo preclinical model (PCa12), derived from a patient and partially sensitive to Akt and AR inhibition, was established. Mice were treated with a combination of ipatasertib (50 mg/kg per day), castration, and enzalutamide (10 mg/kg per day), and tumor volumes were monitored until resistance developed (fig. S1D). Upon the emergence of acquired resistance, defined as an increase in tumor volume following initial response, prostate cancer organoids were established from the control tumor and the resistant tumors (Fig. 2A). In vitro cell proliferation studies were performed to demonstrate that the resistant lines were less responsive to Akt (ipatasertib, 500 nM) inhibition compared to controls (Fig. 2A). In our acquired resistant models, we consistently observed an increase in downstream mTOR signaling that was not significantly responsive to upstream Akt (ipatasertib, 500 nM; 4 hours) inhibition (Fig. 2B). The acquired resistance prostate cancer organoids maintained this resistance during a 30-day withdrawal and adding back of ipatasertib (500 nM) (fig. S1, E and F).

Next, we tested whether this was Akt inhibitor dependent by using increasing concentrations of ipatasertib or an allosteric site inhibitor of Akt (MK2206). Median inhibitory concentration (IC_50_) dose-response curves for both ipatasertib and MK2206 demonstrated a shift to the right in a representative resistant line (Fig. 2, C and D). Furthermore, while ipatasertib has known off target effects on S6K at higher concentrations, MK2206 is highly selective for Akt as it binds to the pleckstrin homology (PH) domain rather than the catalytic site (*10*, *17*). In this context, even at high micromolar doses of MK2206, there was no inhibition of downstream mTOR signaling in the resistant model, despite effective upstream Akt inhibition, highlighting the fact that mTOR activity was uncoupled from Akt signaling (Fig. 2E and fig. S1G). To define the role of mTOR in driving resistance in our models, we targeted mTORC1 with RAD001 (100 nM, 4 hours) in combination with ipatasertib (500 nM, 4 hours) and evaluated a catalytic site inhibitor of mTOR (INK128, 100 nM; 4 hours), resulting in inhibition of mTORC1/2. We found that the addition of RAD001 in combination with ipatasertib or INK128 restored sensitivity using cell proliferation as well as on signaling pathway inhibition as readouts (Fig. 2, F and G). Our data demonstrate that resistance to Akt inhibition can be overcome through mTOR inhibition. While resistance can be driven through restoration of downstream mTOR signaling, it is still important to maintain inhibition of both upstream and downstream PI3K pathway signaling.

To validate these findings, we also studied the development of acquired resistance in the AR-dependent CWR22Pc-EP cell line where *PTEN* was knocked out using the CRISPR-Cas9 technology (fig. S2A). We observed that, following acute (3 day) in vivo treatment with ipatasertib (50 mg/kg per day), both upstream Akt targets and downstream mTOR targets displayed decreased phosphorylation (Fig. 2H). However, following prolonged treatment (4 weeks) and the emergence of acquired resistance, mTOR signaling was restored (Fig. 2H). The mechanism leading to restoration of mTOR signaling in this model may be distinct from the PCA12-acquired resistance model as signaling upstream and downstream mTOR has been restored despite ipatasertib still binding to Akt. AR inhibition (repressed expression of FKBP5, STEAP1, and PMEPA1) was maintained throughout the study, indicating that the early emergence of acquired resistance was associated with restored Akt/mTOR and not AR signaling (fig. S2B).

### Whole-genome knockout screen for resistance to Akt inhibition enriches for mechanisms uncoupling mTOR from Akt signaling

**Fig. 3.**
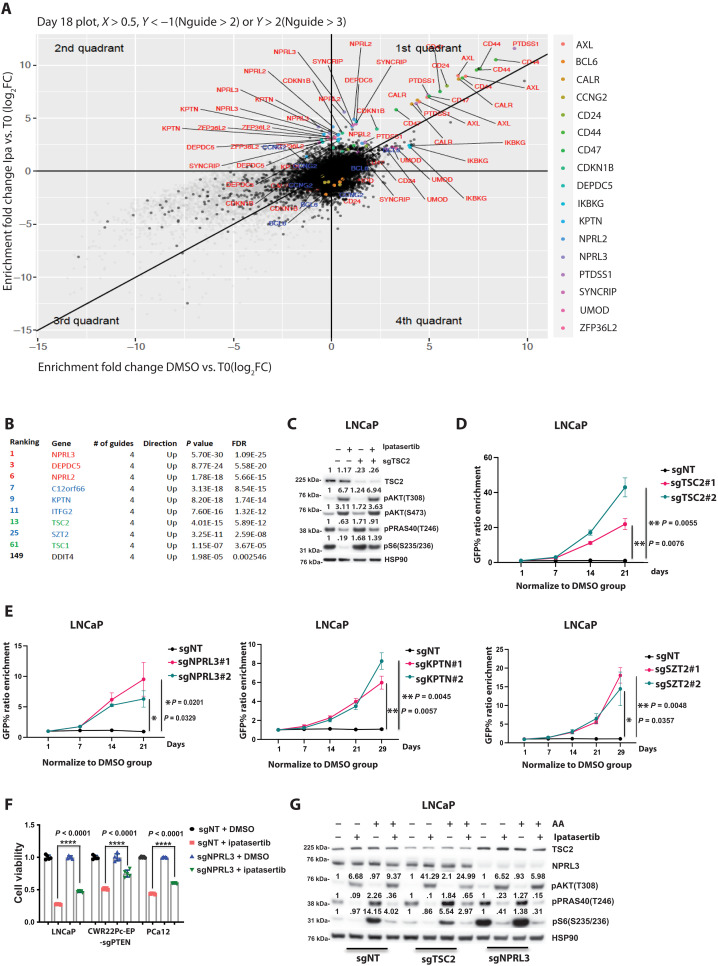
Disrupting the negative regulators of the mTOR pathway, identified as top candidates in a whole-genome knockout screen, enhances resistance to Akt inhibition. (**A**) Plot of the candidate genes from CRISPR-Cas9 knockout (KO) screen in LNCaP cells treated with 450 nM ipatasertib on day 18. (**B**) Top genes identified from the CRISPR-Cas9 KO screen on day 18 (ipatasertib-treated LNCaP cells compared to DMSO-treated cells). (**C**) TSC2-KO LNCaP cells were subjected to a 4-hour treatment with either DMSO or 500 nM ipatasertib. Expression levels of downstream targets in the PI3K pathway were evaluated using Western Blot analysis. (**D** and **E**) Outcomes of a competition assay involving four candidate genes in LNCaP cells. Cells were transduced with lentivirus carrying sgNT-GFP or sgRNA-GFP targeting the candidate genes, each with two specific sgRNAs. Following transduction, cells were treated with either DMSO or 500 nM ipatasertib. The ratio of GFP^+^ cells was normalized to the DMSO treatment group. The data represent the results of three independent experiments (*n* = 3). (**F**) LNCaP, CWR22Pc-EP-sgPTEN, and PCa12, each stably expressing sgNT or sgNPRL3, were subjected to treatment with either ipatasertib (500 nM) or DMSO. Cell viability on day 7 was assessed. (**G**) WT, TSC2-KO, or NPRL3-KO LNCaP cells were exposed to 6 hours of starvation and a 4-hour treatment with either DMSO or 500 nM ipatasertib. Following this, cells were stimulated with or without amino acids for 10 min. The expression levels of downstream targets in the PI3K pathway were evaluated using Western Blot analysis {*P < 0.05, **P < 0.01, and ****P < 0.0001; [(D) to (F)] Welch’s *t* test; error bar represents ±SEM}.

To identify mechanisms driving resistance to Akt inhibition in the context of *PTEN* loss, we took an unbiased approach and performed a genome-wide CRISPR screen in the *PTEN*-deficient cell line LNCaP (fig. S2C). Statistical analyses were performed using false discovery rate (FDR) and *P*-value criteria comparing control dimethyl sulfoxide (DMSO)– versus ipatasertib-treated cells and T0 versus T2 for the individual treatments to select significant enrichment/depletion present in both comparisons. Among the top enriched genes depleted in the ipatasertib-treated condition were negative regulators of mTOR, consistent with the uncoupling of Akt and mTOR activation seen in the derivation of acquired resistance sublines (Fig. 3A). For example, genes composing the tuberous sclerosis complex (TSC) complex (*TSC2* and *TSC1*) were significantly enriched as well as genes encoding for the components of the GATOR1 (*NPRL2, NPRL3, and DEPDC5*) and KICKSTOR (*C12orf66, KPTN, ITFG2, and SZT2*) complexes (Fig. 3B). The TSC complex is the PI3K pathway canonical inhibitory complex of mTOR, where activation of Akt directly phosphorylates TSC2, leading to dissociation of the complex and activation of mTOR activity (*18*–*20*). It is well established that loss of *TSC1* or *TSC2* promotes aberrant mTOR activity, and we hypothesized that this uncoupling of Akt-mTOR signaling would lead to persistent mTOR activation despite Akt being inhibited. To prove this, we knocked out *TSC2* in LNCaP cells and demonstrated that downstream mTOR signaling was significantly less responsive to Akt inhibition (ipatasertib, 500 nM; 4 hours) (Fig. 3C). Furthermore, using an in vitro competition assay, we demonstrated that knockout of TSC2 promoted resistance to the Akt inhibitor ipatasertib (500 nM) (Fig. 3D).

Like the TSC complex, the GATOR1/KICSTOR complex is a negative regulator of mTOR. While the TSC complex is directly regulated by Akt, the GATOR1/KICSTOR complex regulates mTOR activity based on the availability of cellular free amino acids (*21*–*23*). To evaluate whether loss of function of the GATOR1 or KICSTOR complexes resulted in a resistant phenotype, we knocked out *NPRL3*, *KPTN*, or *SZT2* in the LNCaP cell line and demonstrated through competition assays that the loss of these genes drove resistance to Akt inhibition (ipatasertib, 500 nM) (Fig. 3E). We hypothesized that this resistance was driven, in part, by maintenance of downstream mTOR signaling uncoupled from Akt signaling. In support of this, we found that knockout of *NPRL3* across several Akt inhibitor sensitive lines promoted resistance to Akt inhibition on cell proliferation assays and that downstream mTOR signaling was not robustly diminished compared to nontargeting control (Fig. 3F and fig. S2D). To further evaluate the functional implications of *NPRL3* knockout, we evaluated mTOR signaling following amino acid starvation and stimulation (10 min). As expected, knockout of *TSC2* and *NPRL3* demonstrated divergent findings, where, in *TSC2*-deficient cells, amino acid stimulation activated mTOR signaling, and this was not observed following loss of *NPRL3* (Fig. 3G).

**Fig. 4.**
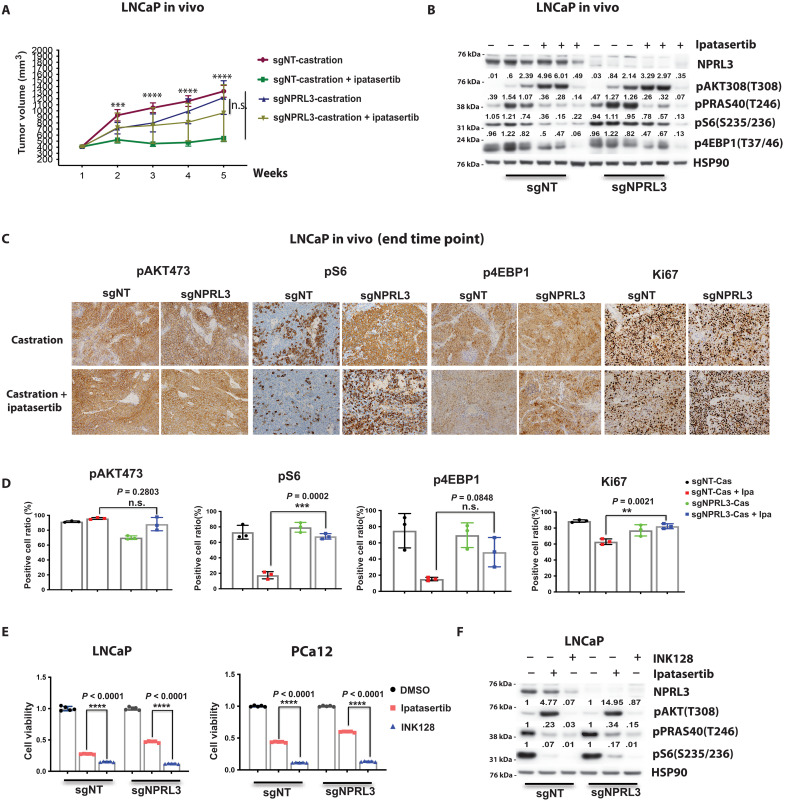
Targeting mTOR directly within NPRL3 knockout contexts restores sensitivity to Akt inhibition. (**A**) Comparative growth of NPRL3-WT and NPRL3-KO LNCaP tumors in NSG mice subjected to ipatasertib treatment (50 mg/kg) or vehicle. Castration was performed when tumors reached ~300 mm^3^. (**B**) Western blot analysis illustrating the status of PI3K signaling in LNCaP-sgNT and Lncap-sgNPRL3 tumors following a 4-week treatment period, as referenced in (A). (**C**) Immunohistochemistry images illustrating the levels of pAkt473, pS6, p4EBP1, and Ki67 in LNCaP-sgNT and LNCaP-sgNPRL3 tumors following a 5-week treatment regimen. (**D**) Quantification of immunohistochemistry data of (C). (**E**) Cell viability analysis on day 7 in LNCaP-sgNT, LNCaP-sgNPRL3, Pca12-sgNT, and Pca12-sgNPRL3 cells treated with ipatasertib (500 nM), INK128 (100 nM), or DMSO. (**F**) LNCaP-sgNT and LNCaP-sgNPRL3 cells were treated with ipatasertib (500 nM), INK128 (100 nM), or DMSO for 4 hours. Cell lysates were subjected to Western blot analysis to assess protein expression levels in PI3K pathway {**P < 0.01, ***P < 0.001, and ****P < 0.0001; n.s., not significant; (A) multiple *t* test and [(D) and (E)] Welch’s *t* test; error bar represents ±SEM}.

To further validate the role of loss of function alterations in the GATOR1/KICSTOR complex in driving resistance to Akt inhibition, we performed in vivo studies using the LNCaP model system with and without *NPRL3* knockout. In support of our screen and in vitro validation studies, we found that knockout of *NPRL3* promoted resistance to combined ipatasertib (50 mg/kg per day) and castration (Fig. 4A). Western blot analysis demonstrated that *NPRL3*-deficient tumors had increased pS6 and more sustained pS6 following Akt inhibition (Fig. 4B). Immunohistochemical studies performed on end of study tumors demonstrated Ki67, pS6, and p4EBP1 staining in a greater percentage of cells in NPRL3 knockout versus control tumors indicative of sustained mTOR signaling (Fig. 4, C and D).

**Fig. 5.**
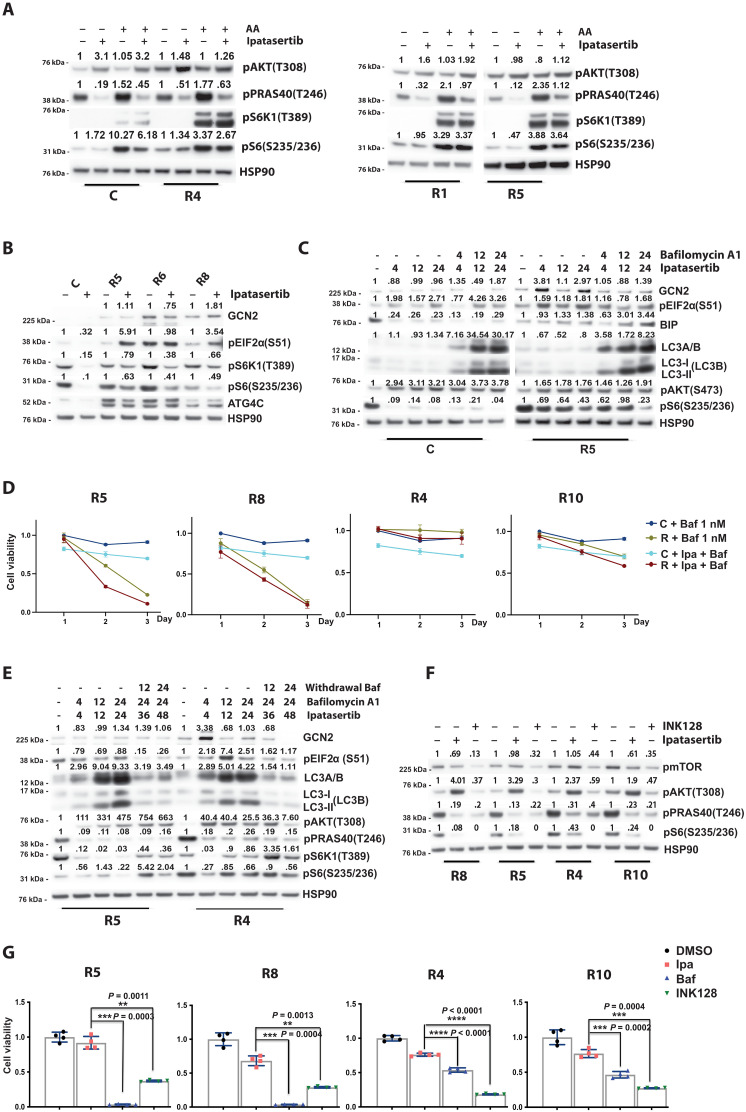
Amino acid sensing is hyper-tuned in resistant models, and autophagy inhibition restores Akt inhibitor sensitivity in a subset. (**A**) C, R4, R1, and R5 cells were starved for 6 hours, followed by a 4-hour treatment with DMSO or ipatasertib (500 nM). Cells were stimulated with or without amino acids for 10 min, and PI3K pathway targets were analyzed by Western blot. (**B**) Western blot analysis of autophagy-related proteins in R5, R6, and R8 cells compared to the control line after treatment with DMSO or ipatasertib (500 nM) for 4 hours. (**C**) Western blot analysis of autophagy targets in control and R5 cells treated with ipatasertib (500 nM), autophagy inhibitor bafilomycin A1 (1 nM), or DMSO for various durations. (**D**) Cell viability was measured over 3 days in ipatasertib-resistant lines (R5, R8, R4, and R10) and control cells treated with bafilomycin A1 (1 nM) or a combination of bafilomycin A1 and ipatasertib (500 nM). (**E**) Western blot of autophagy markers in R5 and R4 cells treated with ipatasertib, bafilomycin A1, or DMSO. Samples were collected at multiple time points, including after bafilomycin A1 withdrawal at 24 hours. (**F**) PI3K/mTOR pathway targets were evaluated by Western blot in resistant lines (R8, R5, R4, and R10) after treatment with INK128 (100 nM), ipatasertib (500 nM), or DMSO for 4 hours. (**G**) Cell viability on day 3 was assessed in resistant lines treated with bafilomycin A1 (1 nM), ipatasertib (500 nM), INK128 (100 nM), or DMSO [**P < 0.01, ***P < 0.001, and ****P < 0.0001; (G) Welch’s *t* test; error bars, ±SEM].

Inhibiting mTOR directly in the context of *NPRL3* knockout with a catalytic site inhibitor of mTOR (INK128, 100 nM; 4 hours), which targets upstream Akt and downstream mTOR activity, restored sensitivity (Fig. 4, D and E). Given that our whole-genome CRISPR knockout screen identified negative regulators of mTOR activation through the nutrient sensing arm, we directly assessed the impact on amino acid stimulation of mTOR in our acquired resistance lines. These experiments revealed that amino acid sensing was hyper-tuned to amino acid stimulation of mTOR signaling but not responsive to Akt inhibition (Fig. 5A).

### mTOR is dependent on endosomal nutrient processing in a subset of Akt inhibitor acquired resistance models

Autophagy is the physiologic process of conserved cellular degradation and recycling of cellular products and nutrients through a lysosome-dependent regulated mechanism to promote cell survival. This process is known to be active during cellular stress caused by nutrient starvation, oncogenic activation, and inhibition of mTOR signaling among others. On the basis of the observation that the acquired resistance models that we developed were hyper-tuned to amino acid stimulation of mTOR signaling, we asked whether endosomal reprocessing of nutrients was playing a role in promoting resistance to Akt inhibition. We examined a subset of our acquired resistance and control organoids and observed an increase in expression of markers associated with autophagy in our ipatasertib-resistant models (Fig. 5B). These acquired resistance organoids demonstrated increased levels of GCN2, ATG4C, and pEIF2α. Similar findings were also observed in our CWR22PC-EP-sgPTEN ipatasertib-resistant tumors (fig. S3A).

To evaluate the role of autophagy and nutrient processing as a mechanism in supporting cell survival and mTOR signaling in our acquired resistant models, we performed assays with the late-stage autophagy inhibitor bafilomycin A1. Treatment with bafilomycin A1 (1 nM) demonstrated a more rapid accumulation of LC3A/B, accumulation of lipidated autophagosome associated LC3B (II), and down-regulation of pS6 in an acquired resistance model (Fig. 5C). Withdrawal of bafilomycin A1 restored pS6 levels and LC3A/B levels to baseline, indicating that the observed phenotype was dependent on the inhibition of autophagy (fig. S3B). Next, we performed in vitro growth assays across a panel of our acquired resistance models to determine whether these models were sensitive to bafilomycin A1 (Fig. 5D and fig. S3C). We discovered that some acquired resistance models were exquisitely sensitive to low nanomolar doses of bafilomycin A1, while others were not, suggesting that autophagy and endosomal reprocessing of nutrients were promoting resistance to ipatasertib in the bafilomycin A1–sensitive models.

To further define this, we evaluated changes in LC3A/B accumulation, LC3B lipidation levels, and mTOR signaling in bafilomycin A1–sensitive (R5) and bafilomycin A1–nonsensitive (R4) prostate cancer organoids over a time-course experiment (Fig. 5E). In the bafilomycin A1–sensitive line (R5), addition of bafilomycin A1 to ipatasertib repressed downstream mTOR signaling that was restored following bafilomycin A1 withdrawal. This was not observed in the bafilomycin A1–nonsensitive line (R4). Across our bafilomycin A1–sensitive and bafilomycin A1–nonsensitive acquired resistance organoids, we can illustrate that all these models demonstrate sensitivity to mTORC1/2 inhibition (INK128, 100 nM) in biochemical and in vitro growth assays, highlighting the critical role of mTOR downstream signaling in promoting resistance to Akt inhibition (Fig. 5, F and G).

Collectively, our data demonstrate that restoration of mTOR signaling is a key mechanism in driving resistance to Akt inhibition, and this can be achieved through multiple genetic and adaptive nutrient regulating processes. In the setting of Akt inhibitor resistance, therapeutic efficacy can be restored by inhibiting mTOR with agents directly targeting mTOR or through the inhibition of autophagy and nutrient reprocessing.

## DISCUSSION

Over the past decade, the therapeutic landscape for the clinical management of patients with high risk locally advanced and metastatic prostate cancer has markedly evolved (*16*). While effective targeting of the AR and androgen axis remains the cornerstone of therapy, several precision-based therapies have been developed on the basis of the molecular alterations enriched in prostate cancer. This is evident by the clinical success of poly(adenosine 5′-diphosphate–ribose) polymerase inhibition in breast cancer type 1/2 susceptibility (*BRCA1/2*)-altered prostate cancers and the exceptional response to checkpoint inhibitors in MSH (MutS Homolog)–altered prostate cancers (*24*, *25*). A phase 3 trial of olaparib treatment for men with progressive mCRPC harboring DNA damage response pathway alterations demonstrated a significant improvement in radiographic progression-free survival compared to placebo (*25*). In addition, for *PTEN*-deficient prostate cancers, small-molecule inhibitors of Akt combined with second-generation AR pathway inhibition have demonstrated efficacy in mCRPC (*7*). The phase 3 IPATential150 trial demonstrates several important findings (*7*). First, patients harboring loss of PTEN had significantly lower response to second-generation AR inhibition, validating the preclinical studies, demonstrating that loss of PTEN drives resistance to AR inhibitors (*4*, *5*). Second, combined Akt and AR inhibition resulted in a significant benefit on radiographic progression-free survival for PTEN-deficient tumors. Last, genomic evidence of PI3K pathway alterations was a more robust predictor of response to combined Akt and AR inhibition compared to criteria defining loss of PTEN in ≥50% of cells on immunohistochemical assays.

While our initial discovery laid the foundation for the development of clinical trials evaluating combined PI3K and AR pathway inhibition in prostate cancer, our current work highlights the diversity of responsiveness among prostate cancers harboring loss of *PTEN* and further defines mechanisms of response and resistance. We discovered through preclinical modeling of acquired resistance that the dominant mechanism driving resistance to combined Akt and AR inhibition is the restoration of downstream mTOR signaling. We show that sensitivity to Akt inhibition could be restored with the addition of agents targeting mTOR.

In accordance with our acquired resistance modeling, the loss of negative regulators of mTOR was enriched in our whole CRISPR screen to define mechanisms of resistance to Akt inhibition. This included the canonical PI3K-regulated complex TSC as well as the GATOR1/KICSTOR complexes that regulate mTOR signaling in response to amino acid availability. The loss of these regulatory complexes uncouples Akt signaling from mTOR, allowing persistent mTOR signaling despite Akt inhibition. Our current work is consistent with a previous study in ER-positive *PIK3CA*-mutant breast cancer that identified aberrant mTOR signaling as a dominant mechanism of resistance to PIK3CA-targeted therapy (*26*). Like our acquired resistance models, resistance driven by loss of these complexes could be overcome by targeting mTOR.

While we did not observe any molecular alterations in the TSC or GATOR1/KICSTOR complexes in our acquired resistance models, we found that mTOR signaling was hyper-tuned to amino acid stimulation. Furthermore, markers of autophagy were up-regulated in our acquired resistance models, suggesting that cellular nutrient and amino acid recycling were playing a role in restoring mTOR signaling. Inhibition of mTOR is known to activate autophagy through a process regulated by translation and transcription (*27*–*29*). Autophagy supports cell survival during stress and recycles critical cellular needs. We found that, in a subset of our acquired resistance models, restoration of mTOR signaling was dependent on autophagy and the recycling of cellular nutrients. These models were dependent on autophagy for cell survival and proliferation.

Collectively, our data demonstrate that there are multiple mechanisms leading to restoration of mTOR signaling as a driver of resistance to Akt inhibition. This has important clinical implications as the addition of an mTOR inhibitor or inhibition of the autophagic process could restore sensitivity to Akt inhibition.

## METHODS

### Patient-derived prostate cancer organoids and cell lines

Patient-derived organoids (PDOs) with the designations MSK-PCa1, MSK-PCa2, MSK-PCa3, MSK-PCa8, MSK-PCa11, MSK-PCa12, MSK-PCa15, and MSK-PCa16 were generated according to previously described methods (table S1) (*9*). The acquisition of human tissue for prostate cancer organoids was conducted under Memorial Sloan Kettering Cancer Center (MSKCC) Institutional Review Board–approved protocol nos. 06-107 and 12-001. PDOs were cultured and maintained under standard organoid culture conditions, as previously described (*9*).

The CWR22Pc-EP cell line was generated and maintained following established protocols (*30*). The LNCaP cell line (ATCC CRL-1740) and CWR22Pc-EP cell lines were cultured in RPMI medium supplemented with 10% fetal bovine serum (Omega, FB-11). *PTEN* was knocked out in Pca15 and Pca16 organoids, as well as in CWR22Pc-EP cells, as documented in the published paper (*31*).

We established a set of ipatasertib-resistant cell lines denoted as R1 to R12, alongside a control line referred to as C, derived from the Pca12 model. Control line C was derived from xenografts in mice subjected to castration and enzalutamide treatment and then cultured in a medium containing 500 nM enzalutamide. In contrast, all the ipatasertib-resistant lines (R1 to R12) were generated from mice subjected to castration, enzalutamide, and ipatasertib treatment and, subsequently, cultured in a medium supplemented with 500 nM enzalutamide and 500 nM ipatasertib. All cell lines and prostate cancer organoids used in our studies have tested negative for mycoplasma with the Myplasma Detection Kit (Lonza).

### Therapeutic agents

The Akt inhibitor ipatasertib, provided by Genentech, was dissolved in a solution consisting of 0.5% methylcellulose and 0.2% Tween 80 for use in our in vivo study. AR inhibitor enzalutamide was synthesized by the MSKCC chemistry core. We acquired MK2206 (S1078), PD0325901(S1036), Gefitinib (S1025), INK128 (S2811), and RAD001 (S1120) from Selleck Chemicals. Bafilomycin A1 (0.1 mg/ml in DMSO) was obtained from Thermo Fisher Scientific (J67193XF).

### Immunoblot

We generated cell lysates using radioimmunoprecipitation assay buffer, separated the proteins on NuPAGE Novex 4 to 12% Bis-Tris Protein Gels, and transferred them onto polyvinylidene difluoride membranes. Following blocking with 5% bovine serum albumin in tris-buffered saline and Polysorbate 20 (TBST), primary antibodies were applied overnight at 4°C, succeeded by secondary antibodies at room temperature for 1 hour. If samples were processed on different blots, then we ensured consistency by developing signals for all samples simultaneously. All immunoblots were conducted with three independent biological replicates.

The following antibodies were used for Western blotting: PSA (5365S), pAkt (Ser^473^) (Cell Signaling Technology, 4060L), pAkt (Thr^308^) (Cell Signaling Technology, 4056S), pEGFR (Cell Signaling Technology, 3777S), p-p44/42 MAPK (Erk1/2) (Thr^202^/Tyr^204^) (D13.14.4E) XP (Cell Signaling Technology, 4370S), pMEK1/2 (Ser^217/221^) (41G9) (Cell Signaling Technology, 9154S), pS6K (Thr^389^) (Cell Signaling Technology, 9205L), pS6 (Ser^235/236^) (Cell Signaling Technology, 4856S), PTEN (Cell Signaling Technology, 9188L), pPRAS40 (Thr^246^) (Cell Signaling Technology, 2997S), p4EBP1 (Thr^37/46^) (Cell Signaling Technology, 2855L), pmTOR (Ser^2448^) (Cell Signaling Technology, 5536S), TSC2 (Cell Signaling Technology, 4308S), HSP90 (Cell Signaling Technology, 4875S), GCN2 (Cell Signaling Technology, 3302S), pEIF2α (Ser^51^) (Cell Signaling Technology, 3398S), LC3A/B (Cell Signaling Technology, 12741S), LC3B (Cell Signaling Technology, 277S), β-actin (Cell Signaling Technology, 4970S), NPRL3 (Thermo Fisher, PA5-52849), ATG4C (Cell Signaling Technology, 5262S), BIP (Cell Signaling Technology, 3177S), AR (Abcam, AB108341), FKBP5 (Cell Signaling Technology, 12210S), STEAP1 (Santa Cruz Biotechnology, SC-271872), and PMEPA1 (Santa Cruz Biotechnology, SC-293372).

### CellTiter-Glo–based cell viability assay

In each well of a 96-well plate, a range of 0.5 × 10^3^ to 1 × 10^3^ of human organoids, 2 × 10^3^ CWR22Pc-EP, or 5000 × 10^3^ LNCaP were seeded in 100 μl of medium. The following day, drugs were introduced to each well, bringing the total volume to 200 μl. After 72 hours, both the media and drugs were replenished to ensure continued efficacy. On the seventh day, the viability of the cells was assessed using the CellTiter-Glo Luminescent Cell Viability Assay kit (Promega, G7573) in accordance with the provided protocol. All cell proliferation assays were repeated with at least two independent biological replicates.

### RNP-mediated CRISPR genome editing

Cas9 nuclease was combined with 1.2 μM single-guide RNA [sgRNA; Integrated DNA Technologies (IDT)] and incubated for 10 to 20 min to create Cas9-ribonucleoprotein (cRNP) complexes. Dissociated organoid cells, ranging from 0.5 to 1 million, were suspended in nucleofection buffer (Lonza kit R, VVCA-1001) along with the cRNP complexes and electroporation enhancer (IDT, 1075915), for a total volume of 100 μl. The cell suspension was transferred to a nucleofection cuvette and electroporated using the Lonza Amaxa Nucleofector II (Program X-005). Following electroporation, the cells were centrifuged and seeded for subsequent culturing (*32*). sgRNA target sequence: NT (nontargeting, human), ACGGAGGCTAAGCGTCGCAA; NPRL3, TAGACGT-TGTTCTCACACAG; TSC2, CAGCATCTCATACACACGCG; EGFR1, CTGCGTGATGAGCTGCACGG; and EGFR2, GAGAACCTAGA-AATCATACG.

### Mouse xenograft procedure

We implanted a range of 2 × 10^6^ to 5 × 10^6^ cells subcutaneously into male NSG mice aged between 6 to 8 weeks (the Jackson Laboratory, no. 005557). Castration was performed once the tumor volume reached ~200 to 300 mm^3^. In the treatment group, mice received ipatasertib orally at a dosage of 50 mg/kg per day and enzalutamide at 10 mg/kg per day from Monday to Friday. Tumor sizes were measured weekly, and all experimental procedures strictly adhered to the approved Institutional Animal Care and Use Committee (IACUC) protocol 06-07-012.

### Whole-genome CRISPR-Cas9 knockout screen

The screening used the Human CRISPR Brunello Knockout Library. Lentiviruses carrying the sgRNA library were introduced to 560 million LNCaP cells at a multiplicity of infection (MOI) of 0.33, ensuring 1000× coverage of the library. Subsequently, the library-transduced cells were exposed 18-day treatment period with either DMSO or ipatasertib (495 nM). Experiments were conducted in collaboration with our gene editing and screening core.

FASTQ preprocessing was performed using FastX Toolkit to generate a counts file. Filtering was applied to exclude sgRNAs and samples with low counts. Guides that did not have greater than five counts per million were excluded. A minimum of two counts was required across all experimental samples, and a count per million greater than 5 in two or more samples was required. Enriched genes were identified using the Bioconductor package edgeR_3.42.4. Enrichment analysis at the guide level was done using R package 4.1.0.

Guide enrichment data from day 18 at both DMSO and ipatasertib condition were organized and ordered by fold change (log_2_) and *P* value, based on statistical comparison to guide count at T0. We are specifically interested in CRISPR guides whose depletion is due to Ipatasertib treatment but not those who target general essential genes. We identified guides targeting essential genes by their significantly depletion in DMSO versus T0 condition (FDR < 0.25). These guides were excluded for further analysis. We further filtered the gene list by the number of their guides detected (total of four guides per gene) (DMSO, *n* > 3; and ipatasertib, *n* > 2). The final enrichment guide list was generated by plotting an intersection of guides with enrichment fold change (log_2_FC) > 2 in ipatasertib versus T0 condition and those log_2_FC > −1 in DMSO versus T0 condition. The final depletion guide list was generated by plotting an intersection of guides with depletion fold change (log_2_FC) < −1 in ipatasertib versus T0 condition and those logFC> −0.5 in DMSO versus T0 condition. Ggplot2 was used to generate enrichment/depletion *x*-*y* plot. *X* axis shows enrichment fold change (log_2_) in DMSO versus T0 condition. *Y* axis shows enrichment fold change (log_2_) in Ipatasertib versus T0 condition. Enriched guides (first quadrant) were labeled in red. Depleted guides (third quadrant) were labeled in blue. Nontargeting guides were labeled in black.

### Competitive cellular growth validation assay

The Cas9 expression construct (Addgene, 52962) was transfected into 293 T cells using Lipofectamine 2000 (Invitrogen, 11668-500) to produce lentiviruses. LNCaP cells were then infected with the lentivirus for 48 hours and selected with Blasticidin (10 μg/ml) for 4 to 5 days. These LNCaP-Cas9 cells were transduced with lentiviruses containing either sgNT–green fluorescent protein (GFP) or sgRNA-GFP, targeting specific genes, with a MOI between 20 and 50%. Three days after infection, the cells were stained with 4′,6-diamidino-2-phenylindole, and the GFP ratio was measured using flow cytometry (T0). The cells were then treated with either DMSO or 500 nM ipatasertib, and the GFP ratio was monitored by flow cytometry at 7-day intervals.

### Quantification of immunoblot results

Immunoblot analyses were quantified using Fiji. Each protein band was measured and first normalized to its corresponding control band (e.g., HSP90 or β-actin). In addition, all treated samples were normalized to the control sample.

### Quantification of immunohistochemistry slides

Slides were scanned with Pannoramic Flash (3DHistech, Hungary) using 20×/0.8 numerical aperture objective lens. Regions of interest were then drawn and exported as .tif files from these scans using Slideviewer (3DHistech, Hungary). These images were then imported into ImageJ/Fiji [National Institutes of Health (NIH), USA] to apply color deconvolution to separate the hemotoxylin and 3,3′-diaminobenzidine (DAB) signals. Thresholding and area measurement were used to determine the total DAB-positive area and tissue area. Watershedding of the hematoxylin was used to segment the cells, and percentage area of DAB staining was used to get a count of the cells with positive staining.

### Study approval

All experiments involving animals were approved by MSKCC Animal Ethics Committee (IACUC protocol 06-07-012).
