# Supplementary material for: Uncoupling of Akt and mTOR signaling drives resistance to Akt inhibition in PTEN loss prostate cancers
Source: Sci Adv. 2025 Feb 7;11(6):eadq3802. doi: 10.1126/sciadv.adq3802 (PMC11804928; doi:10.1126/sciadv.adq3802)
Supplement: Supplementary file 1 — Figs. S1 to S3 Table S1 Legends for data S1 and S2 [file sciadv.adq3802_sm.pdf]

Science Advances
AAAS

Supplementary Materials for

Uncoupling of Akt and mTOR signaling drives resistance to Akt inhibition in PTEN loss prostate cancers

Ninghui Mao *et al.*

Corresponding author: Brett S. Carver, carverb@mskcc.org

Sci. Adv. **11**, eadq3802 (2025)
DOI: 10.1126/sciadv.adq3802

The PDF file includes:

Figs. S1 to S3
Table S1
Legends for data S1 and S2

Other Supplementary Material for this manuscript includes the following:

Data S1 and S2

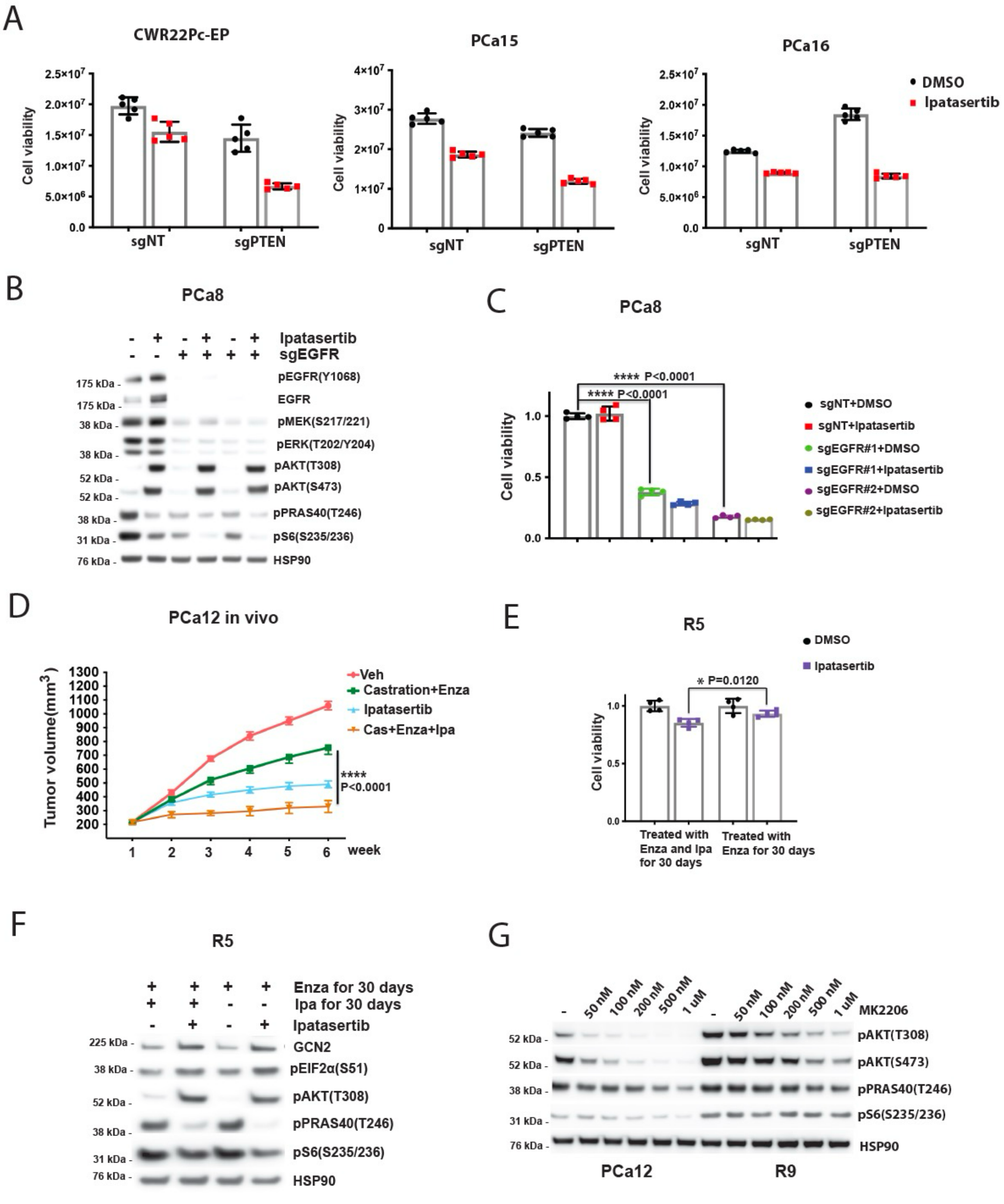

A
CWR22Pc-EP
PCa15
PCa16
Cell viability
sgNT
sgPTEN
DMSO
Ipatasertib
B
PCa8
Ipatasertib
sgEGFR
pEGFR(Y1068)
EGFR
pMEK(S217/221)
pERK(T202/Y204)
pAKT(T308)
pAKT(S473)
pPRAS40(T246)
pS6(S235/236)
HSP90
C
PCa8
**** P<0.0001
**** P<0.0001
sgNT+DMSO
sgNT+Ipatasertib
sgEGFR#1+DMSO
sgEGFR#1+Ipatasertib
sgEGFR#2+DMSO
sgEGFR#2+Ipatasertib
D
PCa12 in vivo
Tumor volume(mm3)
Veh
Castration+Enza
Ipatasertib
Cas+Enza+Ipa
****
P<0.0001
week
E
R5
DMSO
Ipatasertib
* P=0.0120
Treated with Enza and Ipa for 30 days
Treated with Enza for 30 days
F
R5
Enza for 30 days
Ipa for 30 days
Ipatasertib
GCN2
pEIF2α(S51)
pAKT(T308)
pPRAS40(T246)
pS6(S235/236)
HSP90
G
50 nM
100 nM
200 nM
500 nM
1 uM
MK2206
pAKT(T308)
pAKT(S473)
pPRAS40(T246)
pS6(S235/236)
HSP90
PCa12
R9

Supplemental Fig.1. EGFR VIII expression in Pca8 contributes to resistance against AKT inhibition.

(**A**) Cell viability on day 7 in PTEN WT and CRISPR PTEN-modified CWR22Pc-EP, PCa15, and PCa16 treated with either ipatasertib (500 nM) or DMSO was assessed. (**B**) EGFR KO Pca8 cells were subjected to a 4-hour treatment with either DMSO or 500 nM ipatasertib. Expression levels of downstream targets in the PI3K and MAPK pathway were evaluated using Western Blot analysis. (**C**) PCa8 expressing sgNT or sgEGFR were subjected to treatment with either ipatasertib (500 nM) or DMSO. Cell viability on day 7 was assessed. **(D)** Comparative growth of Pca12 tumors in NSG mice treated with enzalutamide (10 mg/kg), ipatasertib (50 mg/kg), or vehicle. Castration was performed when tumors reached 200~300 mm^3. (**E**) The cell viability on day 7 was assessed in the ipatasertib-resistant cell line R5. The resistant cells were treated with enzalutamide (500 nM) alone or in combination with ipatasertib (500 nM) for a duration of 30 days before the assay. (**F**) The Western Blot depicts the PI3K signaling for samples obtained from Panel S1E. **(G)** The expression levels of downstream targets in the PI3K pathway were evaluated in both PCa12 and R9 using Western Blot analysis. Cells were treated with DMSO or various doses of AKT inhibitor MK2206 for 4 hours. (*$p<0.05$, ****$p<0.0001$, C, D, E: Welch's t test, error bar represents ±SEM)

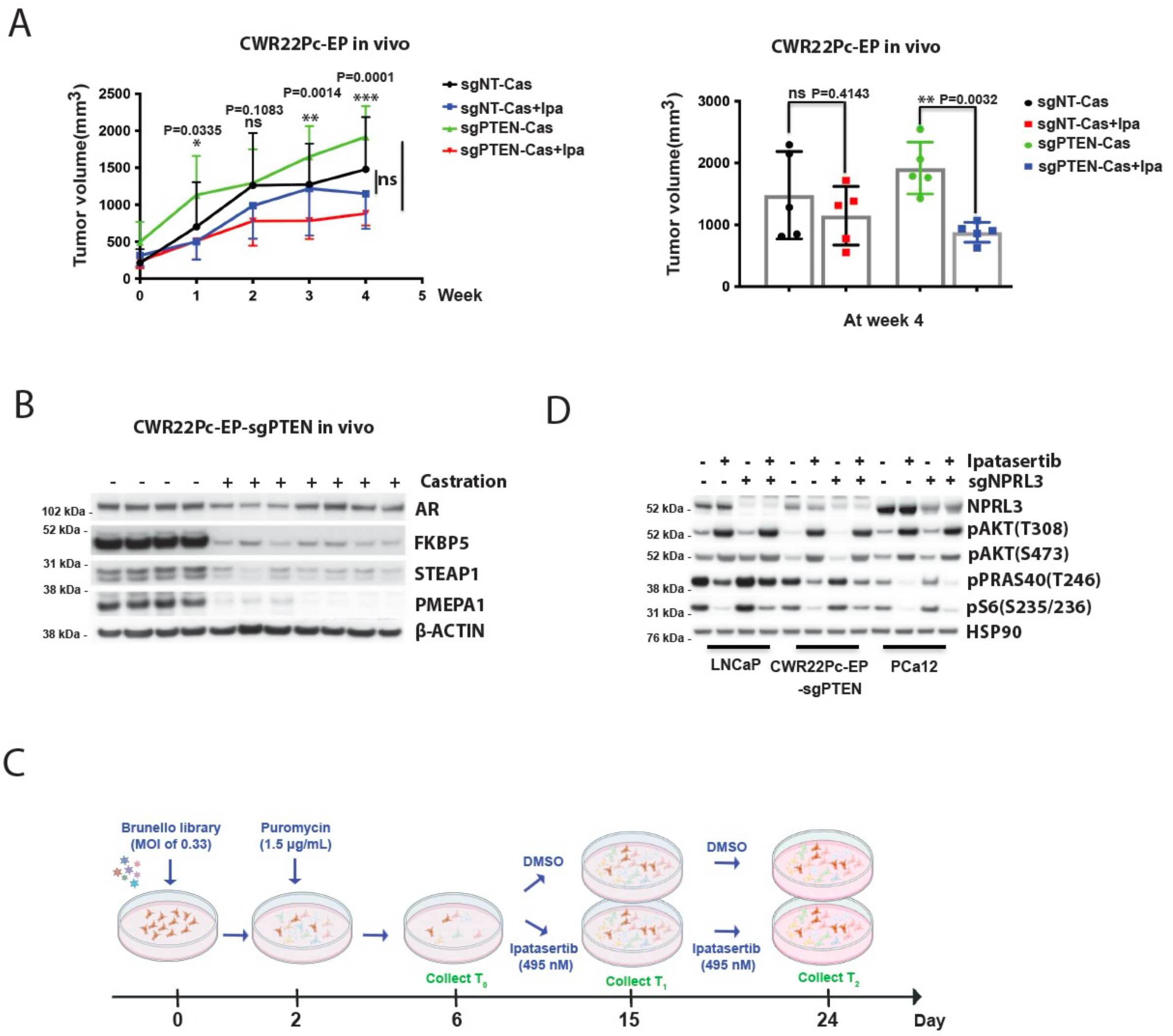
A
CWR22Pc-EP in vivo
Tumor volume(mm3)
2500
2000
1500
1000
500
0
0 1 2 3 4 5 Week
P=0.0335
*
P=0.1083
ns
P=0.0014
**
P=0.0001
***
ns
sgNT-Cas
sgNT-Cas+Ipa
sgPTEN-Cas
sgPTEN-Cas+Ipa
CWR22Pc-EP in vivo
Tumor volume(mm3)
3000
2000
1000
0
ns P=0.4143
** P=0.0032
At week 4
sgNT-Cas
sgNT-Cas+Ipa
sgPTEN-Cas
sgPTEN-Cas+Ipa
B
CWR22Pc-EP-sgPTEN in vivo
- - - - + + + + + + +
Castration
102 kDa - AR
52 kDa - FKBP5
31 kDa - STEAP1
38 kDa - PMEPA1
38 kDa - β-ACTIN
D
- + - + - + - + - + - +
Ipatasertib
- - + + - - + + - - + +
sgNPRL3
52 kDa - NPRL3
52 kDa - pAKT(T308)
52 kDa - pAKT(S473)
38 kDa - pPRAS40(T246)
31 kDa - pS6(S235/236)
76 kDa - HSP90
LNCaP
CWR22Pc-EP -sgPTEN
PCa12
C
Brunello library (MOI of 0.33)
Puromycin (1.5 µg/mL)
DMSO
Ipatasertib (495 nM)
DMSO
Ipatasertib (495 nM)
Collect T0
Collect T1
Collect T2
0
2
6
15
24
Day

Supplemental Fig.2. Schematic Representation of the CRISPR Screening Process

(A) The growth curve illustrates the progression of PTEN-WT and PTEN-KO CWR22Pc-EP tumors in NSG mice subjected to either ipatasertib treatment (50mg/kg) or vehicle. Castration intervention was initiated when tumors reached approximately 300mm³. **(B)** Western Blot analysis depicting the modulation of androgen receptor targets in CWR22Pc-EP-sgPTEN tumors after 30-day castration. **(C)** LNCaP cells transduced with the Human CRISPR Brunello Knockout Library (1000X coverage) were treated for 18 days with either DMSO or ipatasertib (495 nM). Cells were collected in triplicates at T0, T1 (day 9), and T2 (day 18). **(D)** Western Blot analysis illustrating PI3K signaling in sg-NPRL3 samples derived from panel 3F. (***$p<0.001$, **$p<0.01$, *$p<0.05$, n.s: not significant, A: multiple t test and Welch's t test, error bar represents ±SEM).

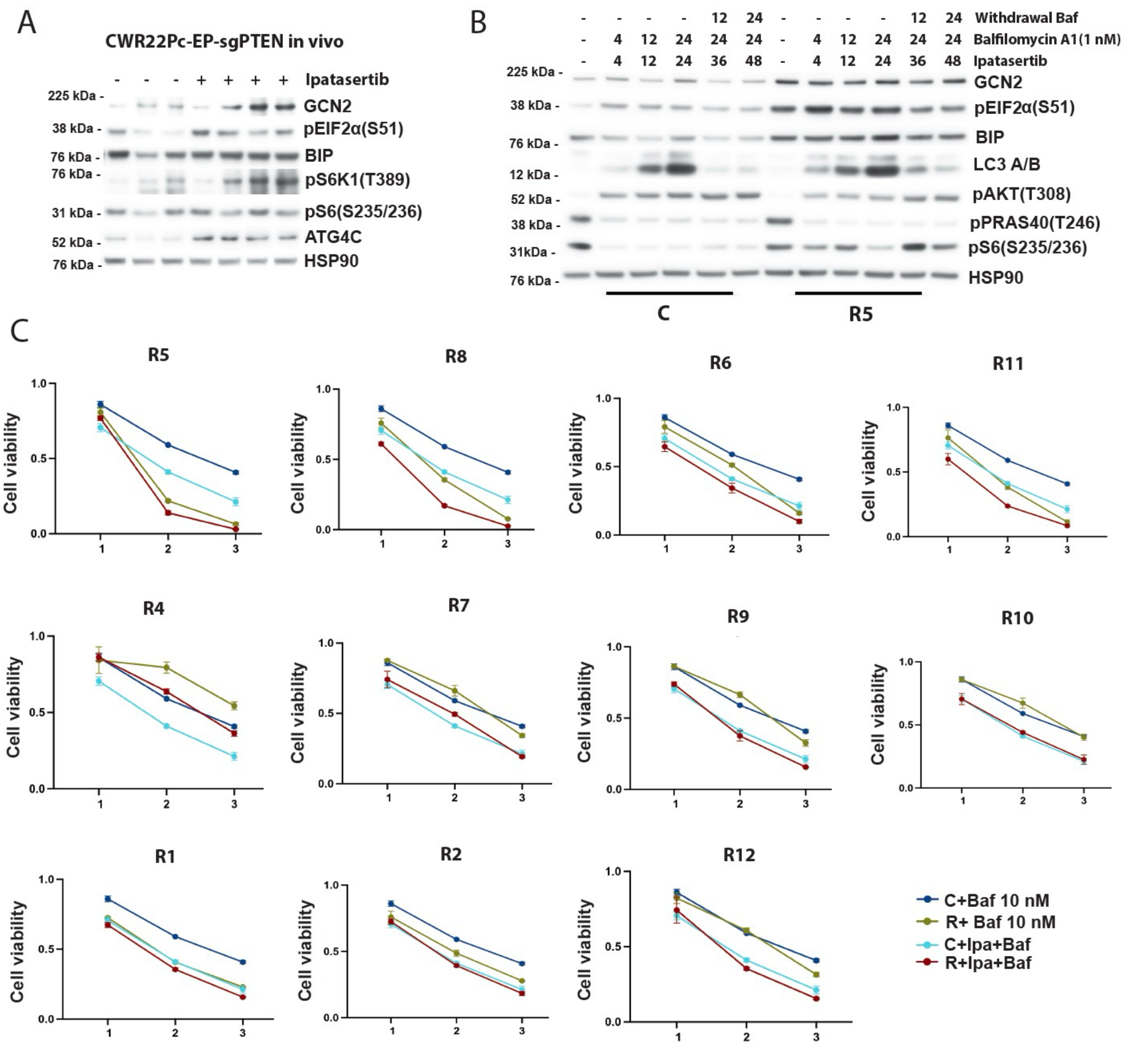
A
CWR22Pc-EP-sgPTEN in vivo
Ipatasertib
GCN2
pEIF2α(S51)
BIP
pS6K1(T389)
pS6(S235/236)
ATG4C
HSP90
B
Withdrawal Baf
Balfilomycin A1(1 nM)
Ipatasertib
GCN2
pEIF2α(S51)
BIP
LC3 A/B
pAKT(T308)
pPRAS40(T246)
pS6(S235/236)
HSP90
C
R5
C
R5
R8
R6
R11
R4
R7
R9
R10
R1
R2
R12
Cell viability
C+Baf 10 nM
R+ Baf 10 nM
C+Ipa+Baf
R+Ipa+Baf

Supplemental Fig.3. There is a differential sensitivity to autophagy inhibition in acquired resistant models.

(A) Western blot analysis of autophagy-related targets in PTEN-KO CWR22Pc-EP tumors from panel S2A. **(B)** Western blot analysis depicts the expression levels of autophagy-related targets in both control and R5 cell lines. Cells underwent treatments with 500 nM ipatasertib, 1 nM Bafilomycin A1, or DMSO at various time points. Furthermore, two time points were examined, reflecting the withdrawal of Bafilomycin A1 after 24 hours of treatment. **(C)** Cell viability assessment over a three-day period in ipatasertib-resistant cell lines (R5, R8, R6, R11, R4, R7, R9, R10, R1, R2 and R12) and the control line. Cells were subjected to different treatments: 10 nM Bafilomycin A1 alone or in combination with 500 nM ipatasertib.

Pre-clinical Model	AR	PTEN	TP53	PIK3CA	Histology	ARi Sensitivity	AKTi Sensitivity
**CWR22PC**	++	Wild-type	Wild-type	Mutant	Adenocarcinoma	Sensitive	Partial
**LNCaP**	++	Loss	Wild-type	Wild-type	Adenocarcinoma	Sensitive	Sensitive
**PCa1**	--	Loss	Mutant	Wild-type	Adenocarcinoma	Resistant	Sensitive
**PCa2**	++	Loss	Mutant	Wild-type	Adenocarcinoma	Sensitive	Sensitive
**PCa3**	+	Loss	Wild-type	Wild-type	Adenocarcinoma	Resistant	Resistant
**PCa8**	+	Loss	Mutant	Wild-type	Adenocarcinoma	Partial	Resistant
**PCa11**	+	Loss	Mutant	Wild-type	Adenocarcinoma	Partial	Partial
**PCa12**	+	Loss	Mutant	Wild-type	Adenocarcinoma	Partial	Sensitive
**PCa15**	+	Wild-type	Mutant	Wild-type	Adenocarcinoma	Partial	Partial
**PCa16**	--	Wild-type	Mutant	Wild-type	Adenocarcinoma	Resistant	Partial

Supplemental Table.1. Pre-clinical models description.

Table 1 shows the AR expression (++ for normal/high, + for weak, -- for absent), genetic status of PTEN, TP53, PIK3CA, histologic classification, and responsiveness to AR and AKT inhibition for the individual parental models used across our experiments.

Supplemental data.1. Top genes identified from the CRISPR-Cas9 knockout screen on day 18 (ipatasertib-treated LNCaP cells compared to DMSO-treated cells)

Supplemental data.2. Top genes identified from the CRISPR-Cas9 knockout screen on day 18 (ipatasertib-treated LNCaP cells compared to T0 cells).
